# Glassy Magnetic Behavior and Correlation Length in Nanogranular Fe-Oxide and Au/Fe-Oxide Samples

**DOI:** 10.3390/ma12233958

**Published:** 2019-11-29

**Authors:** L. Del Bianco, F. Spizzo, G. Barucca, G. Marangoni, P. Sgarbossa

**Affiliations:** 1 Dipartimento di Fisica e Scienze della Terra, Università di Ferrara, I-44122 Ferrara, Italy; federico.spizzo@unife.it; 2 Dipartimento SIMAU, Università Politecnica delle Marche, I-60131 Ancona, Italy; g.barucca@staff.univpm.it; 3 Dipartimento di Ingegneria Industriale, Università di Padova, I-35131 Padova, Italy; giovanni.marangoni@unipd.it (G.M.); paolo.sgarbossa@unipd.it (P.S.)

**Keywords:** disordered magnetism, super-spin glass, glassy correlation length, magnetic freezing, nanogranular Au/Fe-oxide, Fe-oxide nanocrystallites

## Abstract

In nanoscale magnetic systems, the possible coexistence of structural disorder and competing magnetic interactions may determine the appearance of a glassy magnetic behavior, implying the onset of a low-temperature disordered collective state of frozen magnetic moments. This phenomenology is the object of an intense research activity, stimulated by a fundamental scientific interest and by the need to clarify how disordered magnetism effects may affect the performance of magnetic devices (e.g., sensors and data storage media). We report the results of a magnetic study that aims to broaden the basic knowledge of glassy magnetic systems and concerns the comparison between two samples, prepared by a polyol method. The first can be described as a nanogranular spinel Fe-oxide phase composed of ultrafine nanocrystallites (size of the order of 1 nm); in the second, the Fe-oxide phase incorporated non-magnetic Au nanoparticles (10–20 nm in size). In both samples, the Fe-oxide phase exhibits a glassy magnetic behavior and the nanocrystallite moments undergo a very similar freezing process. However, in the frozen regime, the Au/Fe-oxide composite sample is magnetically softer. This effect is explained by considering that the Au nanoparticles constitute physical constraints that limit the length of magnetic correlation between the frozen Fe-oxide moments.

## 1. Introduction

Magnetic systems classifiable as ‘disordered’ have the common property that the constituent magnetic moments undergo, at a critical temperature, a collective freezing along essentially random directions, giving rise to a low-temperature quasi-degenerate frozen state. This phenomenology requires basic ingredients, which are topological disorder, mixed and competing magnetic interactions, and random local anisotropy.

For instance, in amorphous magnetic materials the atomic spins may give rise to a non-collinear magnetic structure (speromagnetic or asperomagnetic) as a result of the competition between magnetic anisotropy and exchange interaction and depending on the distribution in sign of the exchange coupling constants [1].

In dilute magnetic alloys of noble metals (e.g., Au, Ag, Cu, and Pt) with 3d transition metal impurities (e.g., Fe or Mn), the randomness of site occupancy of the atomic spins and the frustration of competing magnetic interactions (RKKY and dipolar) result in a canonical spin glass behavior, characterized by the transition from a high-temperature paramagnetic state of the spins to a low-temperature frozen regime [2,3,4]. In the spin glass description, an important parameter is the length on which the spins are rigidly coupled together under the action of competitive magnetic interactions [5]. In a canonical spin glass, this magnetic correlation length increases more and more on reducing temperature across the freezing one, and in principle, it reaches an infinite extension in the final, low-temperature frozen regime. It is worth mentioning that, depending on the specific system, the frozen regime can also develop out of a ferromagnetic state of the spins (re-entrant behavior) [5,6,7,8].

The advent of nanoscience has led to the creation of engineered magnetic materials (nanoparticles, nanogranular materials, nanocrystalline thin films, and multilayers), showing novel magnetic effects of huge relevance in strategic technological sectors (e.g., energy, spintronics, data storage, sensors, biotechnology, and nanomedicine). This intense research activity on nanostructured magnetic systems has also disclosed the existence of a variety of disordered magnetism phenomena.

It is now quite well established that a random assembly of dipolar interacting magnetic nanoparticles may undergo a collective freezing, resulting in a low-temperature glassy magnetic regime [9,10,11,12,13,14,15,16,17]. The frozen regime is often termed “super-spin glass” (SSG), in order to stress that the magnetic entities that undergo the freezing process are not atomic spins, but the nanoparticle moments [18,19,20].

Canting and glassy dynamics of surface spins were observed in ferrite [21,22,23,24,25] and antiferromagnetic nanoparticles [26,27], as a result of reduced atomic coordination and altered super-exchange bonds. A spin glass like freezing was reported for the spins located at the grain boundaries of ball-milled antiferromagnetic FeRh [28], pure nanocrystalline Fe [29], and Fe thin films [30], due to the combination of structural disorder and distributed (in magnitude and sign) exchange interactions.

Disordered magnetism effects were found to dominate the magnetic properties of two-phase nanogranular systems. For instance, pellets obtained by the high-pressure compaction of core-shell Fe/Fe-oxide nanoparticles were described as being composed of a structurally disordered Fe-oxide matrix embedding Fe nanoparticles [31]. The magnetic study revealed a magnetic freezing of net Fe-oxide moments, a behavior which was termed “cluster-glass like.” In nanogranular samples consisting of Ni nanoparticles embedded in a NiO matrix, the presence of a structurally disordered NiO components at the interface between the ferromagnetic (FM) and antiferromagnetic (AFM) phases and showing a glassy magnetic behavior, ruled the magnetic properties of the whole composite system [32]. In materials of this type, the observation of the exchange bias effect allowed valuable information to be obtained on the disordered magnetism phenomenology. It is worth recalling that the exchange bias effect is the horizontal shift of the hysteresis loop that may be observed in nanostructured FM/AFM systems after field-cooling through the Néel temperature of the AFM, so that the AFM spins couple to the FM ones, minimizing the interface exchange interaction [33,34]. The loop shift reveals the existence of a unidirectional anisotropy for the FM spins, due to the torque action exerted on them by the AFM ones. It was found out that the role of the AFM in this exchange coupling mechanism may also be played by a glassy magnetic phase whose magnetization dynamics ends up ruling the magnetic behavior of the whole composite system [35,36,37,38,39,40].

Due to the strategic importance of the exchange bias effect in the technology of spintronic devices [41,42], the exchange coupling mechanism is mainly investigated in FM/AFM samples in the form of films or nanopatterned structures [43]. Disordered magnetism phenomena emerge also in systems with this configuration [44,45,46,47,48,49]. For instance, in NiFe/IrMn bilayers, the existence of a structurally disordered IrMn region with spin glass like behavior, interposed between the NiFe layer and the bulk of the IrMn layer, well accounted for the thermal evolution of the exchange bias effect and for its disappearance at a temperature much lower than expected, namely, much lower than the Néel temperature of the AFM [46]. However, for this glassy phase, the onset of an infinite magnetic correlation length below the freezing temperature was not experimentally observed. This was explained considering that the interface region was inherently inhomogeneous, both from the structural and magnetic point of view, and that this hindered the formation of an infinite correlated frozen state [50]. In the case of NiFe/IrMn nanodots, a dependence of the exchange bias effect on the ratio between the dot size and the glassy correlation length was demonstrated [50,51]. Moreover, it was shown that the exchange bias effect of NiFe/IrMn bilayers could be tuned by varying the correlation length in the IrMn spin glass like region, through the insertion of non-magnetic nanosized Cu elements at the FM/AFM interface [52].

Thus, these findings indicate that a modulation of the correlation length in a glassy magnetic material may be achieved by controlling the structural and/or compositional features. Despite the described extensive research work on the disordered magnetism phenomenology, this specific topic has rarely been addressed. In order to better elucidating it, suitable samples would be needed, with a dominant and well detectable glassy magnetic nature. Composite materials like those described above, in which the glassy magnetic phase is interfaced to a FM one or interposed at the FM/AFM interface, are not particularly suitable, actually. In fact, it is almost invariably observed that, above the freezing temperature, the spins in the FM and AFM components exert a polarizing action on the thermally fluctuating spins of the disordered component, influencing their relaxation dynamics and preventing their passage to the (super)-paramagnetic regime (effect reminiscent of the re-entrant behavior) [29,32,50].

In this context, we have succeeded in preparing two nanogranular samples with peculiar structural properties, extremely favorable for the appearance of disordered magnetism effects. The first is made of ultrafine spinel Fe-oxide nanocrystallites, with sizes of the order of 1 nm. The second sample consists of the same Fe-oxide phase and of Au nanoparticles of 10–20 nm in size. We will show that both samples possess an SSG magnetic character and that the freezing of the Fe-oxide nanocrystallite moments occurrs following a very similar dynamics. However, in the frozen regime, the Au/Fe-oxide composite exhibits a softer magnetic behavior. This effect is discussed considering how the presence of the Au nanoparticles affects the glassy state and highlighting the role of the magnetic correlation length.

## 2. Experimental

### 2.1. Synthesis of the Fe-Oxide and Au/Fe-Oxide Nanogranular Samples

The samples are synthesized by a polyol method [53]. All reagents and solvents are purchased from Sigma-Aldrich and used without further purification.

To prepare the Fe-oxide sample, a solution, obtained dissolving 2.0 g of iron(III) chloride hexahydrate (FeCl_3_·6H_2_O) in 5.0 mL of ethylene glycol and 1.5 mL of water, is injected quickly and under vigorous stirring in 25 mL of an oleylamine (10 g) solution in ethylene glycol, at 190 °C in inert atmosphere (nitrogen). The reaction mixture is heated at reflux for 6 h. After cooling at room temperature, the black suspension is poured into 200 mL of acetone to precipitate the Fe-oxide phase, which is separated magnetically, washed several times with acetone (3 × 50 mL), and dried under vacuum.

The Au/Fe-oxide sample is obtained by introducing the gold precursor into the previously prepared Fe-oxide. To this end, 10 mg of the Fe-oxide phase is dispersed in 1 mL of octylamine and 5 mL of ethylene glycol under magnetic stirring, and heated at 150 °C. The black suspension is then treated with 1 mL of gold(III) chloride hydrate (HAuCl_4_, 30 mg) solution in ethylene glycol, followed by 100 µL of a 50% solution of tetramethylammonium hydroxide in water. The dark-reddish suspension is stirred at room temperature for 45 min and then poured into 250 mL of acetone for magnetic separation. After washing with acetone (4 × 25 mL), the solid precipitate is dried under vacuum.

The Fe-oxide and the Au/Fe-oxide samples are in powder form and labelled MNP and AuMNP, respectively.

### 2.2. Characterization Techniques

The inner structure of the MNP and AuMNP samples is investigated by transmission electron microscopy (TEM) techniques by using a Philips CM200 microscope (Philips, Amsterdam, The Netherlands) operating at 200 kV and equipped with a LaB6 filament. For TEM observations, a small quantity of powder is dispersed in ethanol and subjected to ultrasonic agitation for approximately one minute. A drop of suspension is deposited on a commercial TEM grid covered with a thin carbon film; finally, the grid is kept in air until complete evaporation of ethanol.

The magnetic properties of the samples are investigated by a superconducting quantum interference device (SQUID) magnetometer (Quantum Design, San Diego, USA) operating in the range 5–300 K (maximum applied field H_appl_ = 50 kOe, sensitivity 10^−7^ emu). The powder to be measured is inserted into a suitable holder and slightly pressed to avoid any movement during the measurement.

## 3. Results

### 3.1. Structural Properties Investigated by TEM

The morphology and structure of the MNP and AuMNP samples are analyzed by TEM techniques.

**Figure 1:**
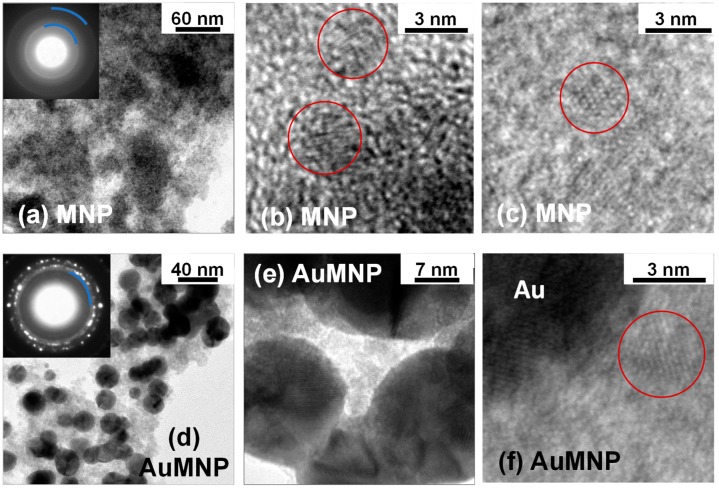
Sample MNP: (**a**) bright field TEM image and corresponding SAED pattern (inset; the blu lines mark the diffraction rings discussed in text); (**b**,**c**) HR-TEM images showing ultrafine Fe-oxide nanocrystallites (red circles). In particular, the circle in frame (**c**) encloses a small Fe-oxide nanocrystallite in [114] zone axis. Sample AuMNP: (**d**) bright field TEM image and corresponding SAED pattern (inset); (**e**) HR-TEM image showing Au nanoparticles in contact with each other and with the Fe-oxide phase; (**f**) HR-TEM image showing a portion of a gold nanoparticle and an ultrafine Fe-oxide nanocrystallite (red circle).

A bright-field TEM image of MNP is shown in Figure 1a. The powder is composed of large agglomerates, which appear as a quite compact medium. Regions with different thickness (with respect to the electron beam direction), consequently providing a different contrast, can be distinguished.

By increasing the magnification and performing high-resolution (HR) TEM observations, it turns out that the sample structure is characterized by a high degree of structural disorder. Typical HR-TEM images, such as those shown in Figure 1b,c, reveal the presence of randomly oriented crystals of about 2–5 nm in size (circled regions). Thus, we model the sample as a nanogranular material made of ultrafine nanocrystallites. The distance between the fringes highlighted in Figure 1b is estimated by the fast Fourier transform (FFT) of the image, performed by using the Gatan Microscopy Suite GMS3 software [54]. The value obtained is d = (0.251 ± 0.05) nm, compatible with the (311) atomic planes of the spinel Fe-oxide structure.

Due to the TEM experimental error, it is not possible to distinguish between magnetite Fe_3_O_4_ (in which d_(311)_ = 0.253 nm) and maghemite γ-Fe_2_O_3_ (d_(311)_ = 0.252 nm). The selected area electron diffraction (SAED) pattern of the sample region imaged in Figure 1a is shown in the inset of the same figure. It features broad and diffuse diffraction rings, confirming the poor crystallinity of MNP. The interplanar distances associated to the two most visible rings (evidenced in the image) are d_1_ = 0.25(2) nm and d_2_ = 0.14(8) nm, corresponding to the family of planes (311) and (440) of the spinel Fe-oxide structure, respectively.

Passing to sample AuMNP, a typical bright field TEM image is shown in Figure 1d. The Fe-oxide phase, which gives the light-grey contrast, incorporates black, roughly spherical elements with sizes between 10 and 20 nm, which correspond to Au nanoparticles. The nature of these two phases is confirmed by SAED measurements. In fact, the SAED pattern (inset of Figure 1d) shows well-defined and speckled diffraction rings, consistent with a crystalline Au phase composed of large grains; in the inner part of the pattern, a diffuse diffraction ring is visible (marked in the figure), which is attributed to the spinel Fe-oxide structure; in particular, to the (311) family of planes.

The HR-TEM analysis further confirms the good crystallinity of the Au nanoparticles, whereas the Fe-oxide phase has a poorly crystalline structure, perfectly similar to that observed in MNP (Figure 1e–f). In most cases the Au nanoparticles are in contact with each other and act so as to spatially confine the Fe-oxide phase, as shown in Figure 1e.

### 3.2. Magnetic Properties

#### 3.2.1. Hysteresis Loops

**Figure 2:**
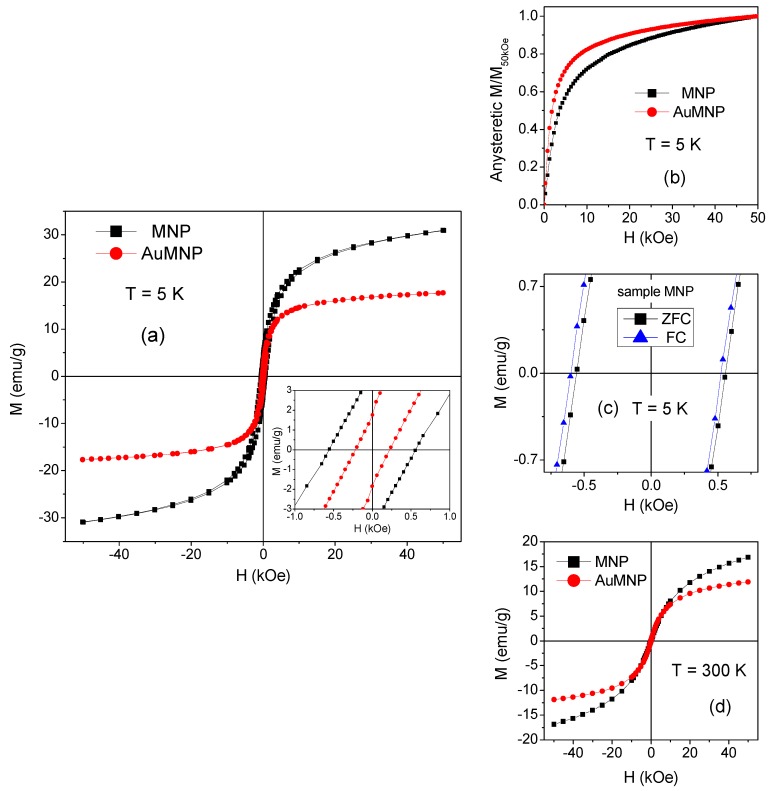
(**a**) Magnetic hysteresis loops measured on the MNP and AuMNP samples at T = 5 K. Inset: close-up of the central region of the loops. (**b**) Anhysteretic magnetization curves for MNP and AuMNP obtained from the loops in (**a**); they are shown as normalized to their value at H = 50 kOe (M_50kOe_). (**c**) Central region of the hysteresis loops measured on sample MNP in zero-field-cooling (ZFC) and field-cooling (FC) mode. (**d**) Magnetization curves measured on MNP and AuMNP at T = 300 K.

Magnetic hysteresis loops measured on MNP and AuMNP at T = 5 K are shown in Figure 2a. In particular, the specific magnetization M is reported, obtained by normalizing the magnetic moment to the mass of the sample. The loops show a non-saturating tendency, more pronounced in MNP. The values of the saturation magnetization M_S_ at T = 5 K are extrapolated from the loops for 1/H tending to zero and are reported in Table 1. By comparing the M_S_ of MNP with that measured in AuMNP, we estimate that the fractions of Fe-oxide and Au in the latter sample are (55 ± 1) wt.% and (45 ± 1) wt.%, respectively. In that calculation, the diamagnetic contribution of Au to the total magnetization is neglected. Since the mass magnetic susceptibility of Au is (−1.42 × 10^−7^) emu/g Oe [55], the diamagnetic contribution to the magnetization at H = 50 kOe is much smaller than the experimental error associated to the M_S_ value. In Table 1, we report the values of coercivity H_C_ and of the irreversibility field H_irr_. The latter parameter is the field at which the ascending and descending branches of the hysteresis loop join together, and it may be considered a measure of the anisotropy field of the system, i.e., H_irr_ = 2 K_eff_/M_S_, where K_eff_ is the effective anisotropy [56,57]. In order to calculate K_eff_ from this relationship, M_S_ must be expressed in (emu/cm^3^); namely, the value of M_S_ for MNP must be multiplied by the density of the Fe oxide phase, which we conventionally take to be 5 g/cm^3^, corresponding to the average of bulk Fe_3_O_4_ and γ-Fe_2_O_3_. The values of K_eff_, also shown in Table 1, and of H_C_, indicate a softer magnetic behavior of sample AuMNP, compared to MNP.

In Figure 2b, we show the anhysteretic magnetization curves, obtained from the loops at T = 5 K and normalized to their value at H = 50 kOe (M_50kOe_), in order to deal with comparable values. A higher initial magnetic susceptibility (i.e., slope dM/dH at low field) and a lower high-field magnetic susceptibility (i.e., slope dM/dH in the field range 30–50 kOe) characterize the anhysteretic magnetization curve of AuMNP, both features indicating that this sample is more easily magnetized, compared to MNP.

Hysteresis loops are also measured at T = 5 K after cooling the samples from room temperature in a field H_appl_ = 50 kOe (field-cooling mode, FC). Compared to the loops in Figure 2a, measured in zero-field-cooling mode (ZFC), the FC loops appear shifted to the left. The effect is shown for sample MNP in Figure 2c, where the central region of the ZFC and FC loops is displayed. The shift can be quantitatively expressed by the positive parameter H_shift_ = −(H_right_ + H_left_)/2, where H_right_ and H_left_ are the points where the loop intersects the field axis. For MNP, H_shift_ = (38 ± 3) Oe, whereas H_shift_ = (11 ± 2) Oe for AuMNP (Table 1).

Hysteresis loops are also measured on the two samples at temperature T = 20, 50, and 300 K. With increasing temperature, H_C_ decreases strongly and no magnetic hysteresis is observed at T = 50 K and 300 K. The loops measured at T = 300 K are shown in Figure 2d.

**Figure 3:**
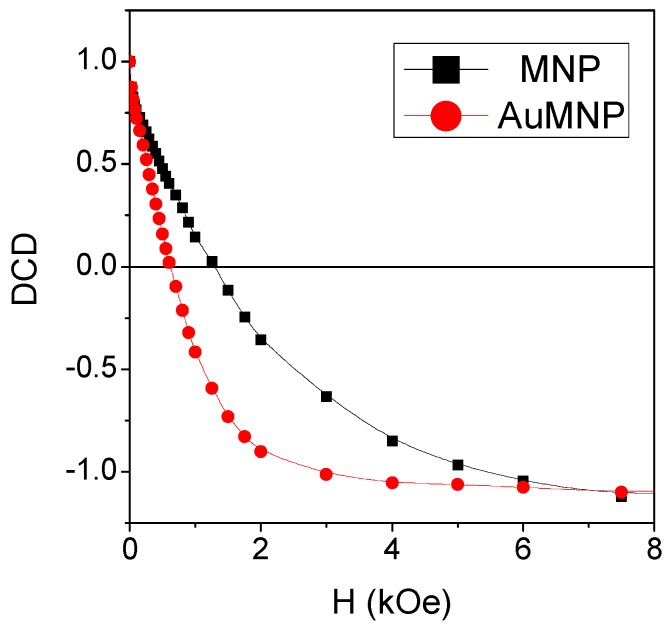
DC demagnetization remanence (DCD) curves, measured at T = 5 K on MNP and AuMNP. The field at which the DCD curve intercepts the x-axis corresponds to the demagnetization remanence coercivity, H_C_DCD_.

The DC demagnetization remanence (DCD) is measured on MNP and AuMNP at T = 5 K, in order to obtain the demagnetization remanence coercivity H_C_DCD_. In the DCD measurement procedure, the sample is initially brought to saturation by a negative magnetic field and then progressively magnetized by a positive field increasing from 0 Oe up to 50 kOe. The recorded remanence values are plotted as a function of the previously applied magnetic field, and the curve so-obtained is normalized to its initial value. The parameter H_C_DCD_ corresponds to the field at which the DCD curve intercepts the x-axis, and therefore, it is a measure of the difficulty of demagnetizing the system [58]. In particular, H_C_DCD_ depends only on the irreversible part of the demagnetization process, unlike the coercivity H_C_, which includes both reversible and irreversible changes. The values of H_C_DCD_, obtained from the DCD curves displayed in Figure 3, are reported in Table 1: the parameter is twice higher in MNP than in AuMNP, which confirms the different magnetic hardness of the two samples at T = 5 K.

#### 3.2.2. Magnetothermal Behavior

**Figure 4:**
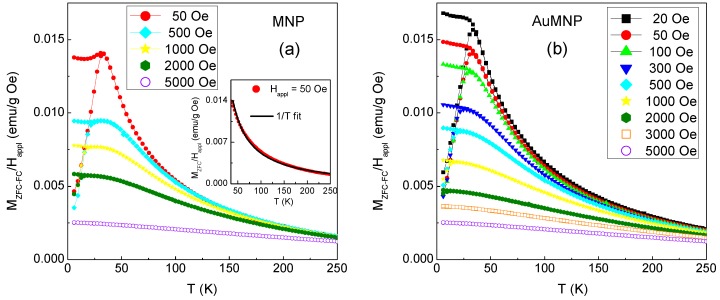
(**a**) Magnetization measured on sample MNP and sample AuMNP (**b**) for increasing temperature (T), at the indicated values of the applied magnetic field H_appl_, after zero-field-cooling (M_ZFC_, lower branch of each displayed curve) and after field-cooling (M_FC_, upper branch). For a better view, the values of M_ZFC_FC_/H_appl_ are displayed, actually. The inset in frame (**a**) shows the M_ZFC_ versus T curve, at H_appl_ = 50 Oe, for T ≥ 35 K (red circular symbols), together with the 1/T fit line (black line).

To gain information on magnetic relaxation processes in the two samples, the magnetization is measured for increasing temperature (heating rate = 3 K/min) in a static magnetic field H_appl_ after cooling the samples from room temperature down to T = 5 K with no applied field (ZFC mode) and in H_appl_ (FC mode). The analysis is carried out at different values of H_appl_, in the 20 Oe–5 kOe range (a higher number of measurements is performed on AuMNP, actually). The curves of M_ZFC_FC_ versus T (each normalized to the respective value of H_appl_) are shown in Figure 4. Their shapes are similar for the two samples. At the lowest values of H_appl_ (20–50 Oe), M_ZFC_ increases on rising temperature from 5 K to about 30 K and then decreases following a 1/T dependence, giving rise to a sharp cusp (in the inset of Figure 4a, the M_ZFC_ versus T curve, at H_appl_ = 50 Oe, is shown for T ≥ 35 K, together with the 1/T fit line). In the same temperature interval, M_FC_ is higher than M_ZFC_, namely, an effect of magnetic irreversibility is visible. The M_ZFC_ and M_FC_ branches join together at the irreversibility temperature T_irr_, which is just slightly higher than that corresponding to the M_ZFC_ peak. A difference between the two samples concerns the trend of the M_FC_ curve in the temperature range where magnetic irreversibility exists. In fact, below T~30 K, M_FC_ in AuMNP increases with decreasing T, whereas the curve of MNP shows a dip, particularly well visible at H_appl_ = 50 Oe.

On increasing H_appl_, T_irr_ reduces as well as the extent of the irreversibility effect. The latter is still visible at H_appl_ = 5 kOe, even if it is not appreciable on the scale of the graph shown in Figure 4, and it is erased at a higher field.

**Figure 5:**
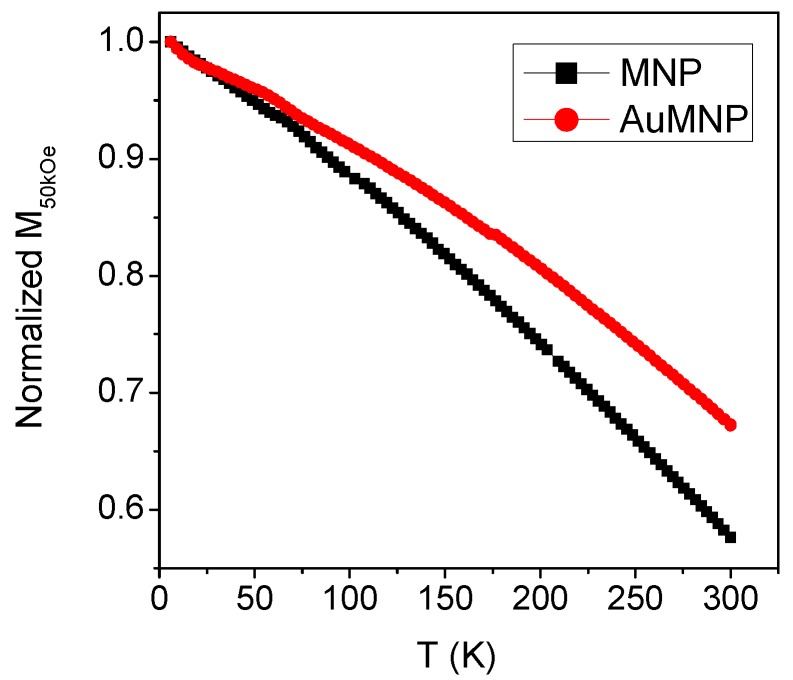
Thermal dependence of the magnetization measured in H_appl_ = 50 kOe (M_50kOe_) on the MNP and AuMNP samples. The curves are normalized to their initial values at T = 5 K.

The magnetization in applied field H_appl_ = 50 kOe (M_50kOe_) is measured on MNP and AuMNP as a function of temperature, in the 5–300 K range (Figure 5). A weaker thermal dependence of M_50kOe_ is observed in AuMNP.

Finally, we report the results of magnetic relaxation measurements carried out on MNP and AuMNP according to the following procedure. The sample is cooled from T = 300 K down to T = 5 K; once this temperature is reached, we allow a 30 s elapse (waiting time, t_w_) before the application of a magnetic field H_appl_ = 50 Oe, and the time variation of the magnetization is recorded. Then, the field is removed, the temperature is raised to 300 K, lowered at T = 5 K, and after t_w_ = 10,800 s (i.e., 3 h), H_appl_ is applied and M versus time is measured again.

**Figure 6:**
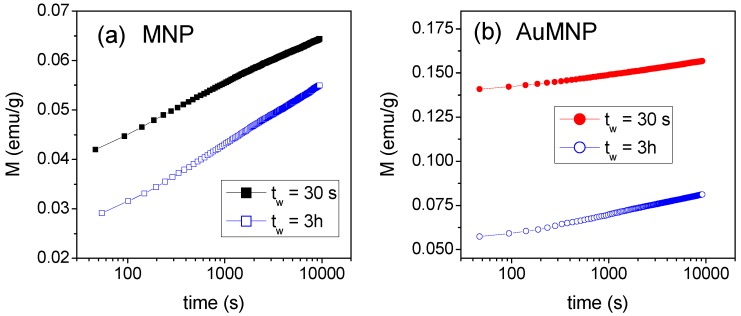
Time dependence of the magnetization measured at T = 5 K, in H_appl_ = 50 Oe, on MNP (**a**) and AuMNP (**b**), for two different waiting times, t_w_ = 30 s and t_w_ = 10,800 s (i.e., 3 h).

The results are shown in Figure 6. Both for MNP and for AuMNP, the two curves are not superposed, which reveals the existence of an aging effect, namely, a dependence of the magnetic relaxation phenomenon on t_w_.

## 4. Discussion

Both the samples investigated show a magnetic behavior typical of the disordered magnetism phenomenology. A clear indication of this is the shape of the M_ZFC-FC_ curves (Figure 4), which is consistent with the onset of a low-temperature frozen collective magnetic regime at T_irr_ (i.e., T_irr_ corresponds to the freezing temperature). This magnetic behavior is certainly prompted by the high structural disorder of the Fe-oxide phase, which is also expected to bring about a marked spin canting effect. In fact, the value M_S_~34.8 emu/g measured at T = 5 K in MNP (Table 1) is definitely lower than that of bulk spinel iron oxide phases (at T = 0 K, Ms~83 emu/g for maghemite and Ms~98 emu/g for magnetite), which reveals a strong lack of spin collinearity [31,59,60]. In this regard, it should be noted that ferrimagnetic iron oxide nanoparticles are often reported to consist of a mix of maghemite and magnetite [61]. The former can appear following the oxidation of the latter, and thus the two phases have a very similar spinel structure, which makes them difficult to be experimentally distinguished.

As observed by TEM (Figure 1), the Fe-oxide phase consists of large agglomerates of randomly oriented, ultrafine nanocrystallites, forming a quite compact, though inhomogeneous, nanogranular material. Hence, the nanocrystallite magnetic moments can magnetically interact both through super-exchange and dipolar interactions, both being ferromagnetic and antiferromagnetic in type. Thus, the following picture can be drawn. At T = 300 K, the magnetic moments are free to thermally fluctuate, i.e., exhibit superparamagnetic relaxation, in line with the absence of magnetic hysteresis (Figure 2d). Evidence that the fluctuating moments at T = 300 K are larger than single atomic spins—which is the reason why we refer to a superparamagnetic state rather than to purely paramagnetic state—is provided by the S-shape of the magnetization curves at this temperature, showing an approach to saturation in the high-field region (Figure 2d). With decreasing temperature, the effective magnetic anisotropy acting on the magnetic moments increases more and more. Below T_irr_, the combination of structural disorder and mix of competing magnetic interactions brings about a collective freezing of the moments along locally varying anisotropy axes, giving rise to a disordered magnetic state, essentially describable as an SSG. The existence of a low-temperature collective frozen magnetic state of the Fe-oxide moments in MNP and AuMNP is definitely confirmed by the observation of the aging effect, i.e., the dependence of the relaxing magnetization on t_w_ (Figure 6). In fact, that is typical of magnetic moment systems governed by glassy dynamics and it reveals the existence of a multidegenerate ground state (multivalley energy structure) [14,35,62,63,64].

This behavior is reminiscent of that shown by the poorly crystalline Fe-oxide phase (mean grain size~2 nm) in the Fe/Fe-oxide system, already recalled in the Introduction (in this respect, it is worth noting that also the value of M_S_ of the Fe-oxide phase was close to that measured in MNP) [31,35]. However, in that case, above the freezing temperature (~20 K), the Fe-oxide moments were not seen to enter the superparamagnetic state, due to the polarizing action of the Fe moments. On the contrary, for MNP and AuMNP, the passage from the frozen to the superparamagnetic regime with increasing temperature above T_irr_ is clearly revealed by the 1/T trend of the M_ZFC-FC_ curves (Figure 4).

**Figure 7:**
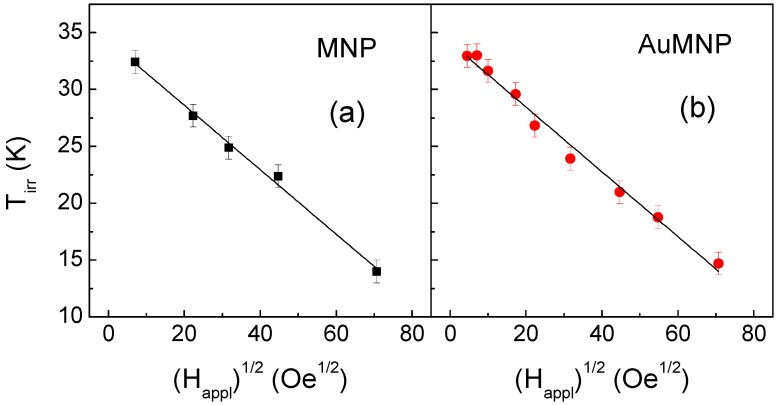
Dependence of the irreversibility temperature T_irr_ on (H_appl_)^1/2^ in MNP (**a**) and AuMNP (**b**). The continuous black lines are the linear fitting curves.

Let us consider these curves in more detail. In canonical spin glasses, T_irr_ follows a (H_appl_)^2/3^ dependence, corresponding to the so-called AT line [65]. In the case of the samples investigated, we find that the evolution of T_irr_ with H_appl_ is better described by a ½ power law dependence. This is shown in Figure 7, where the T_irr_ values are reported as a function of (H_appl_)^1/2^ and the solid lines are the best linear fit. This trend is generally observed in systems where the increase of H_appl_ favors the appearance of a ferromagnetic state, for instance, in re-entrant spin glasses [66]. In our samples, we expect that the Fe-oxide moments align more and more on increasing H_appl_, producing a sort of mean field that adds to H_appl_. This may account for the weaker dependence of T_irr_ on H_appl_, compared to the AT line behavior. As regards the best fit lines in Figure 7, the slope is (−0.28 ± 0.01) K/Oe^1/2^ in both samples and the intercept with the y-axis, which corresponds to T_irr_ for H_appl_ = 0, is (34.3 ± 0.5) K for MNP and (34.2 ± 0.4) K for AuMNP, i.e., is the same within the error. Hence, as far as the analysis of the M_ZFC-FC_ curves is concerned, the two samples are seen to behave in a very similar way.

The horizontal shift of the FC hysteresis loops, observed in both samples and quantitatively expressed by the parameter H_shift_ (Figure 2c), recalls the exchange-bias effect observed in a FM/spin glass system [32,35,67]. When this type of system is field-cooled across the freezing temperature, a spin configuration of the glassy phase is selected through the interface exchange coupling with the FM spins, which in turn favors the FM magnetization to be aligned in the FC direction (unidirectional anisotropy). However, a shift of the FC loop has been also observed in canonical spin glasses, such as the CuMn alloy [68]. The effect is explained considering the presence of chemical inhomogeneities bearing net magnetic moments that freeze along a preferred direction induced by the FC process. Thus, a unidirectional anisotropy arises, which manifests itself in the loop shift. In the case of MNP and AuMNP, the presence of net magnetic moments is intrinsic to their SSG nature, and therefore, the observation of the FC loop shift at T = 5 K is in full agreement with their glassy magnetic behavior.

The pronounced non-saturating tendency of the hysteresis loops measured on MNP and AuMNP at T = 5 K (Figure 2a) and the value of K_eff_ (Table 1)—which, in both samples, is higher than the magnetic anisotropy of bulk magnetite (1.1 × 10^5^ ergs/cm^3^) and maghemite (5 × 10^4^ erg/cm^3^)—are consistent with the collective frozen state of the Fe-oxide moments, implying the existence of high effective anisotropy energy barriers.

The inherent magnetic hardness of the frozen regime is the point on which MNP and AuMNP show their main differences. In fact, K_eff_ is definitely lower in AuMNP than in MNP and the same is true for H_C_ and H_C_DCD_ and H_shift_ (Table 1). The higher initial susceptibility and the lower high-field susceptibility revealed by the anhysteretic curve of AuMNP definitely confirm its reduced magnetic hardness, compared to MNP (Figure 2b). This behavior must be necessarily ascribed to the presence of the Au nanoparticles, which are substantially embedded in the Fe-oxide nanogranular matrix, as shown by TEM (Figure 1d–f). In an ideal spin glass, all the atomic spins that compose the frozen system are magnetically correlated, so as to give rise to a spatially infinite magnetic aggregate [5]. It is to be expected that the same occurs in SSG systems, and therefore, we can schematically hypothesize the existence of an infinite glassy correlation length in MNP. On the contrary, in AuMNP, the Au nanoparticles tend to spatially confine the Fe-oxide phase, and their presence reduces the possibility of direct contact between the magnetic nanocrystallites, hindering the super-exchange coupling and weakening the dipolar magnetic interactions. In other words, in AuMNP, the Au nanoparticles act so as to break the infinite magnetic aggregate, which implies a reduced correlation length, certainly shorter than that in MNP. As a consequence, in AuMNP, the Fe-oxide moments are less firmly frozen and react more easily to the external field, which is in agreement with the lower K_eff_ and with the overall softer magnetic behavior, compared to that of MNP.

Referring again to the M_ZFC-FC_ curves in Figure 4, the dip at very low temperature in the M_FC_ branch of the MNP sample is a feature that can be observed exclusively, but not necessarily, in spin glasses and in SSG systems [69,70]. In AuMNP, you do not actually see it. Based on our description, we infer that the appearance of this feature below T_irr_ is connected to the extension of the glassy correlation length; namely, a slight decrease of the FC magnetic susceptibility in the glassy regime is the hint that largely extended magnetic correlations between the frozen moments are established.

The different trend of M_50kOe_ versus T in MNP and AuMNP (Figure 5) is also attributable to the presence of the Au nanoparticles in the latter sample. In this type of measurement, the sample is subjected to a field H_appl_ = 50 kOe, which erases the low-temperature glassy state and favors the ferromagnetic alignment of the magnetic moments. It is known that the thermal dependence of the magnetization in a saturated ferromagnetic system is due to the collective excitation of the aligned spins [71]. The presence of the Au nanoparticles in AuMNP may modify the spin vibrational modes, compared to those active in MNP. In particular, the thermal dependence of the magnetization is weaker in sample AuMNP, in which the spatial extension of the ferromagnetic order is limited due to the Au nanoparticles.

## 5. Conclusions

We have studied and compared the magnetic behavior of the MNP and AuMNP samples. Both samples possess a glassy magnetic nature. The passage from a high-temperature superparamagnetic regime to a low-temperature SSG regime of the Fe-oxide moments has been revealed by the analysis of the M_ZFC-FC_ versus T curves and by the observation of an aging effect at T = 5 K. The temperature T_irr_, which marks the onset of the freezing process, is similar in the two samples (~34 K, for H_appl_ = 0) as well as its dependence on H_appl_. However, the AuMNP sample is magnetically softer than MNP. This effect has been ascribed to a reduction of the glassy correlation length of the Fe-oxide phase in AuMNP, compared to that in MNP, imposed by the physical constraints represented by the Au nanoparticles.

These findings expand the basic knowledge on disordered magnetism phenomena and glassy magnetic behavior in nanostructured systems, enlightening, in particular, the crucial role of the magnetic correlation length.

## Figures and Tables

**Table 1:** The data refer to the MNP and AuMNP samples, as indicated in column 1, measured at T = 5 K. Column 2: saturation magnetization M_S_. Column 3: Coercivity H_C_. Columns 4 and 5: irreversibility field H_irr_ and effective magnetic anisotropy K_eff_. Column 6. Parameter H_shift_, which quantitatively expresses the shift of the FC loop. Column 7: demagnetization remanence coercivity, H_C_DCD_.

Sample	M_S_ (emu/g) ±1%	H_C_ (Oe) ±1%	H_irr_ (Oe) ±2%	K_eff_ (erg/cm^3^) ±2%	H_shift_ (Oe)	H_C_DCD_ (Oe) 2%
MNP	34.8	559	21300	1.82 × 10^6^	38 ± 3	1300
AuMNP	19.0	221	6300	5.4 × 10^5^	11 ± 2	618
